# Dr. Davis’ Valedictory; Dr. Brainard’s Salutatory

**Published:** 1859-03

**Authors:** 


### EDITORIAL.

### TO THE SUBSCRIBERS FOR THE CHICAGO MEDICAL JOURNAL.

Having resigned my connection with Rush Medical College, and transferred the *Chicago Medical Journal* to Prof. Daniel Brainard, notice is hereby given that all letters, communications, remittances, etc., designed for the *Journal*, should be sent directly to him, he being duly authorized to receive all unpaid subscriptions.

To the many patrons of the *Journal* who have promptly paid their dues, and often added thereto valuable contributions on various topics, we owe many thanks, and hope they will continue the same cordial support to our successor.

N. S. DAVIS.

### TO THE SUBSCRIBERS AND PATRONS OF THE CHICAGO MEDICAL JOURNAL.

Our appearance before you, at the present time, in the capacity of editor, seems to call for some words of explanation, as well as the usual announcement of views and plans for the future.

Circumstances, needless to enumerate, have determined our late colleagues in Rush Medical College, the editors of this journal, to withdraw from that institution. The *Journal* has always been in a degree recognized as the organ of the Faculty, and as an essential part of the system of education planned at the time of its organization. They could not be separated without an essential derangement of these plans, thus far successfully carried out. It became necessary, therefore, for the present editor to assume his former relations to the *Journal*, or relinquish the full consummation of those wishes which have for twenty years been the cherished objects of his life.

This periodical, under the title of *Illinois Medical Journal*, was first issued in the name of Prof. Blaney appearing as editor; but the active co-operation of other members of the Faculty, and the great favor with which it was received by the profession, soon raised it to a high degree of prosperity, and placed it in point of circulation among the first medical journals of this country.

It subsequently passed into the hands of Prof. Evans, under whose efficient editorial charge its popularity was sustained and increased. Since it passed from his hands, its subscription list seems to have been decreased in numbers about one-half, although it still has a wide circulation.

In taking charge of it, the present editor indulges the hope of being able to restore it to its former prosperity. The enterprise, at its first announcement, was regarded as visionary and premature. The country was new, there were no roads, few villages, no cities, and physicians were "few and far between," yet the enterprise met with favor and was crowned with success.

At the present time, everything is changed for the better. Railroads have been built, cities erected, physicians are numerous, and comparatively wealthy. It cannot, therefore, be doubted that the same efforts which first gave value to the *Journal* will restore it to its former state of prosperity.

These efforts will be directed to the making it useful and interesting to its readers, an end to be attained only by publishing what is new and important in a scientific and practical point of view, and excluding all discussions of a personal nature, speculations of a purely theoretical character, and dull, worthless matter generally.

In the department of original articles, arrangements have already been made for securing the assistance of some of the best observers and writers in the State, and the continued aid of all former contributors is most particularly solicited.

For selections, in addition to the exchanges—journals of this country—we have made arrangements for receiving regularly three of the leading journals of Paris, and two of those of London. We expect to complete arrangements for having translations from the German of such articles as are of particular interest.

All valuable medical books, issued from the press in this country, and many of those of Europe, will be noticed in such a manner as to give our readers a just idea of their character and contents, so as to enable each to judge for himself if a work is adapted to his wants.

Finally, items of news, local and foreign, will be carefully gleaned for its pages.

Such is the programme which we offer, and in its fulfilment no pains shall be spared on our part. We cordially invite the aid and co-operation of the patrons of the *Journal*, and all scientific physicians, in efforts to render it valuable and extend its sphere of usefulness.

